# Hepatitis C and Alcohol

**Published:** 2003

**Authors:** Eugene R. Schiff, Nuri Ozden

**Affiliations:** Eugene R. Schiff, M.D., is chief of the Division of Hepatology, director of the Center for Liver Diseases, and a professor of medicine at the University of Miami School of Medicine, Miami, Florida. Nuri Ozden, M.D., is a clinical fellow in hepatology at the University of Miami Center for Liver Diseases, Miami, Florida

**Keywords:** hepatitis C virus, alcoholic beverage, chronic AODE (alcohol and other drug effects), amount of AOD use, epidemiology, risk factors, disease course, alcoholic liver cirrhosis, gender differences, biochemical mechanism, RNA, mutation, apoptosis, inflammation, hepatocellular carcinoma, regulatory proteins, immune response, alcoholic fatty liver, treatment issues, treatment outcome, interferon

## Abstract

Patients infected with the hepatitis C virus (HCV) who drink heavily are likely to suffer more severe liver injury, promoting disease progression to cirrhosis and increasing their risk for liver cancer. Some research, although not conclusive, suggests that even moderate drinking may spur liver damage in HCV-infected patients. Research areas that have the greatest potential for developing more effective treatment options include HCV virology, immunology, animal models, and the mechanisms of liver injury.

Hepatitis C is an infectious liver disease caused by the hepatitis C virus (HCV). The virus, which causes inflammation in the liver and can lead to more serious illness, primarily is spread by intravenous contact with the blood of an infected person. About 4 million people in the United States have been infected, making it the Nation’s most common blood-borne disease, resulting in the deaths of between 10,000 and 12,000 people each year (National Institute of Diabetes and Digestive and Kidney Diseases 2003). Those at heightened risk for HCV infection include intravenous drug users, people who received blood transfusions or organ transplants before 1992 or clotting factors made before 1987, health care workers who suffer needle-stick accidents, and infants born to mothers who are infected with HCV (Centers for Disease Control and Prevention 1998). Several factors may accelerate the progression of hepatitis C, including older age at the time of infection, male gender, obesity, abnormal accumulation of fat in the liver (a condition known as fatty liver, or steatosis), and excessive alcohol consumption (Poynard et al. 2001).

This article discusses the mechanisms by which alcohol may exacerbate HCV-infected patients’ risk of disease progression, reviews issues in the treatment of alcoholic patients with HCV infection, and addresses important areas of future research.

## Epidemiology of Hepatitis C Among Alcoholics

**Figure 1:**
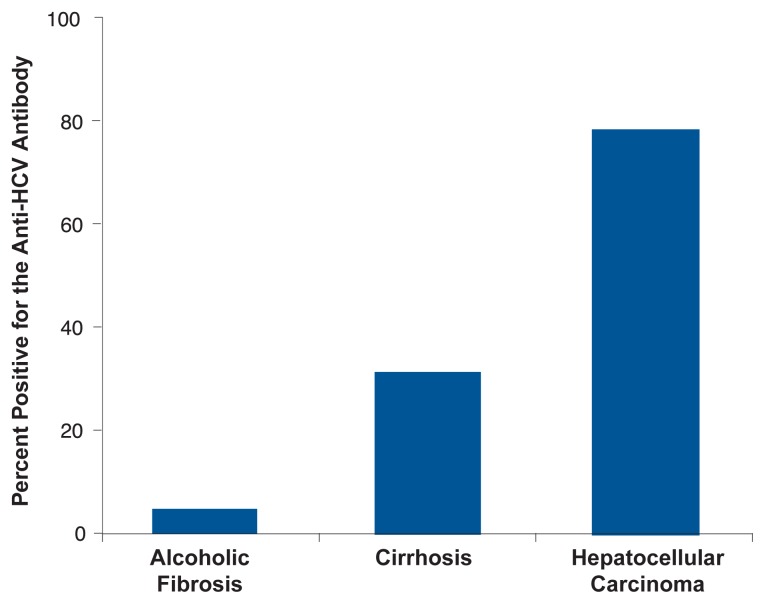
Patients with more severe alcoholic liver disease have a significantly higher prevalence of HCV infection (as assessed by anti-HCV antibody tests). SOURCE: Takase et al. 1993.

Almost one-third of alcoholics with clinical symptoms of liver disease have been infected with HCV, which is four times the rate of HCV infection found in alcoholics who do not have liver disease (Coelho-Little et al. 1995; Mendenhall et al. 1991; Takase et al. 1993). As shown in figure 1, people with more severe liver disease are considerably more likely to test positive for HCV infection than those with less severe liver disease (Takase et al. 1993).

## Levels of Alcohol Consumption in HCV Patients and the Risk of Further Liver Disease

Several studies have indicated that heavy alcohol consumption accelerates patients’ progression from chronic HCV to cirrhosis (a condition in which fibrous scar tissue replaces healthy liver tissue), and liver cancer (specifically, hepatocellular carcinoma, the most common form of liver cancer). Although fewer studies have examined the effects of moderate drinking on the course of liver disease in HCV patients, there is some indication that alcohol consumption in the moderate-to-heavy range may increase HCV-infected patients’ risk of developing liver fibrosis and cirrhosis. Research on whether gender has any effect on alcohol consumption and liver disease progression in HCV patients is very limited.

### Cirrhosis

In a study of 2,235 HCV-infected patients, Poynard and colleagues (1997) observed that patients who drank heavily (more than 50 grams [g] of alcohol, or 4.2 drinks, per day^1^) tended to show much more advanced liver scarring (i.e., fibrosis, a defining feature of cirrhosis) than those who did not drink as heavily. In addition, the researchers identified a group of “rapid fibrosers,” who were more likely to be male, heavy drinkers, and infected with HCV after age 40.

**Figure 2:**
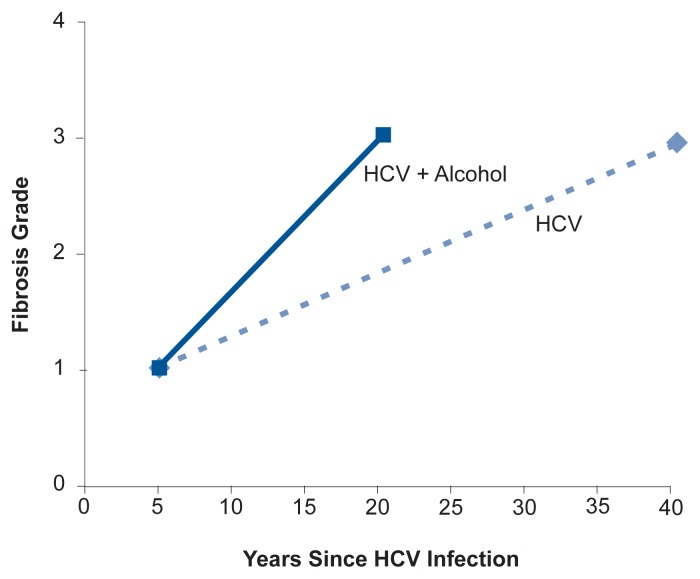
Relationship between fibrosis severity (grades 1–4) and years of HCV infection. Patients who reported significant alcohol consumption (more than 40 g of alcohol per day, or 3.3 drinks, for women; and more than 60 g of alcohol per day, or about 5 drinks, for men) experienced markedly faster disease progression than patients who did not report significant alcohol intake. SOURCE: Wiley et al. 1998.

Wiley and colleagues (1998) observed that HCV-infected patients who drank heavily were significantly more likely to develop cirrhosis than those who were not heavy drinkers. In addition, among patients who did develop cirrhosis, the disease emerged considerably sooner for patients who drank heavily than for those who did not (see figure 2).

Bellentani and colleagues (1999) analyzed hepatitis virus markers (including the virus’s genetic material, HCV RNA, and antibodies to the virus), alcohol intake, and clinical and biochemical evidence of liver disease in a random sample of 6,917 people in northern Italy. Regardless of HCV status, subjects who were heavy drinkers (which the authors defined as drinking more than 30 g, or 2.5 drinks, per day) for more than 10 years were three times as likely to have cirrhosis than those who were not heavy drinkers. Among HCV-positive subjects, 32 percent of those who were heavy drinkers had cirrhosis, compared with 10 percent of those who were not heavy drinkers. There were five cases of hepatocellular carcinoma, all in the heavier drinking group.

Seeff and colleagues (2001) studied 1,030 patients who had been enrolled in a prospective investigation of transfusion-associated HCV conducted in the United States between 1968 and 1980. In a followup investigation conducted an average of 15 years after transfusions had occurred, the researchers found that 17 percent of patients infected with transfusion-associated HCV had developed cirrhosis, compared with 3.2 percent of patients with transfusion-associated non-A, non-B, and non-C hepatitis, and 2.8 percent of uninfected patients. Alcohol use greatly exacerbated patients’ risk of progression from chronic hepatitis to cirrhosis. Patients with both transfusion-associated HCV and a history of heavy alcohol use were 31 times more likely to develop cirrhosis than control subjects without a history of alcohol abuse.

Perhaps the most dramatic demonstration that alcohol multiplies HCV-infected patients’ risk of cirrhosis came from a study by Corrao and Arico (1998). Of 285 cirrhotic subjects, only 1.4 percent (4 subjects) were HCV-positive and nondrinkers, compared with 11.2 percent (32 subjects) who were both HCV-positive and heavy drinkers.

People infected with both HCV and HIV are more likely to develop cirrhosis than are patients who are infected only with HCV, and alcoholism further potentiates this mechanism (Di Martino et al. 2001).

### Liver Cancer

An Italian survey showed that alcohol intake doubles the risk of hepatocellular carcinoma in HCV-infected patients who drink 41 to 80 g of alcohol per day (between 3.4 and 6.7 drinks) and quadruples the risk for patients who drink more than 80 g of alcohol per day (Donato et al. 2002). Other research has shown that HCV-infected patients who drink heavily are more likely to develop liver cancer at a younger age than are those who drink less. For example, Noda and colleagues (1996) observed that patients who drank at least 46 g of alcohol (3.8 drinks) daily were diagnosed with hepatocellular carcinoma an average of 26 years after developing transfusion-associated HCV*,* whereas those who drank less than 46 g per day were diagnosed with cancer an average of 31 years after transfusion. And Kubo and colleagues (1997) found that, among HCV-infected patients with hepatocellular carcinoma, heavy drinkers had more advanced tumors and died sooner after diagnosis than did nondrinkers.

### Moderate-to-Heavy Drinking and Disease Progression

Most studies have evaluated the effect of extremely heavy drinking on the progression of HCV infection to other liver disease, with little emphasis on the effects of moderate drinking on disease progression. However, some studies have found indications that moderate-to-heavy alcohol consumption also may increase the risk of developing liver fibrosis and cirrhosis in patients infected with HCV.

Ostapowicz and colleagues (1998) specified no particular threshold level above which the risk of disease progression increases but found that, among patients with chronic HCV infection, those who had cirrhosis reported higher lifetime and total alcohol consumption than those HCV patients who did not have cirrhosis.

**Figure 3:**
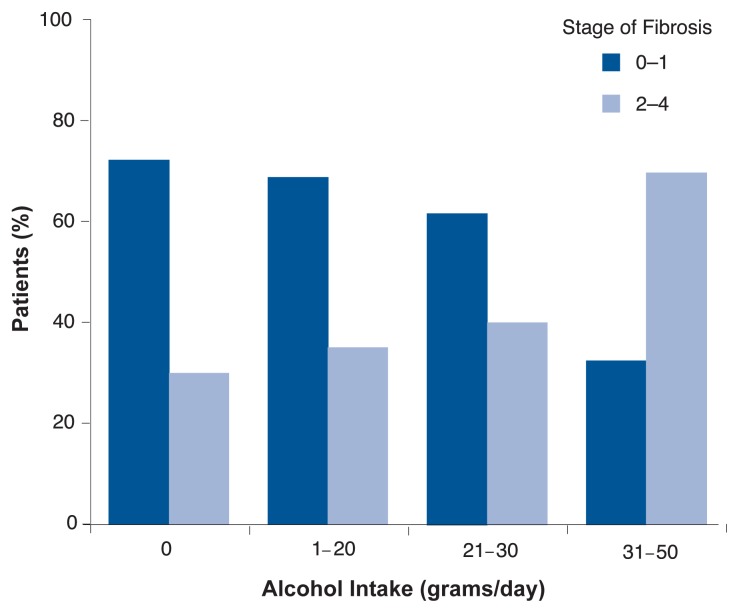
Alcohol consumption and liver fibrosis in patients with chronic HCV. More advanced fibrosis (stages 2 through 4) was observed in 29 percent of patients who reported no alcohol intake, 34.4 percent of those who had minimal intake, 38.2 percent of those who had mild intake, and 67.6 percent of those who were moderate drinkers. Correspondingly, the incidence of less advanced fibrosis (stages 0 through 1) decreased with increasing alcohol consumption. SOURCE: Hézode et al. 2003.

Several other studies have examined the effects of moderate drinking on HCV progression.^2^ Assessing HCV-infected patients who drank less than 40 g of alcohol (3.3 drinks) per day, Westin and colleagues (2002) found that patients whose alcohol intake was above the median level (4.8 g of alcohol, or less than one drink per day) showed more liver scarring (fibrosis) than patients whose alcohol consumption was below the median level. Hézode and colleagues (2003) also found that the severity of fibrosis in patients with chronic HCV was correlated with the amount of alcohol consumed (see figure 3). Finally, a recent study of 180 patients with untreated chronic hepatitis C confirmed these findings (Zarski et al. 2003).

### Gender and Disease Progression

The data regarding the contribution of gender to the progression of HCV in alcoholics is quite limited. Chronic hepatitis C often is milder in women than in men, but women may be more sensitive than men to the adverse effects of alcohol (Becker et al. 1996).

## Histologic Features of Chronic Hepatitis C in Alcoholic Patients

As discussed above, there is strong evidence that heavy alcohol consumption—and perhaps even moderate consumption—increases the risk that HCV infection will progress to more serious liver disease. In patients infected with HCV, alcohol consumption has a direct effect on liver histology. Specifically, patients with HCV who are drinkers show greater liver necrosis, inflammation, fibrosis, and fatty infiltration than HCV patients who do not drink. Further, alcohol consumption and fatty liver have been shown to act together to increase fibrosis in patients infected with HCV, especially in those who are obese and diabetic (Monto et al. 2002).

## Mechanisms of Liver Injury in Alcoholic Hepatitis C Infection

**Figure 4:**
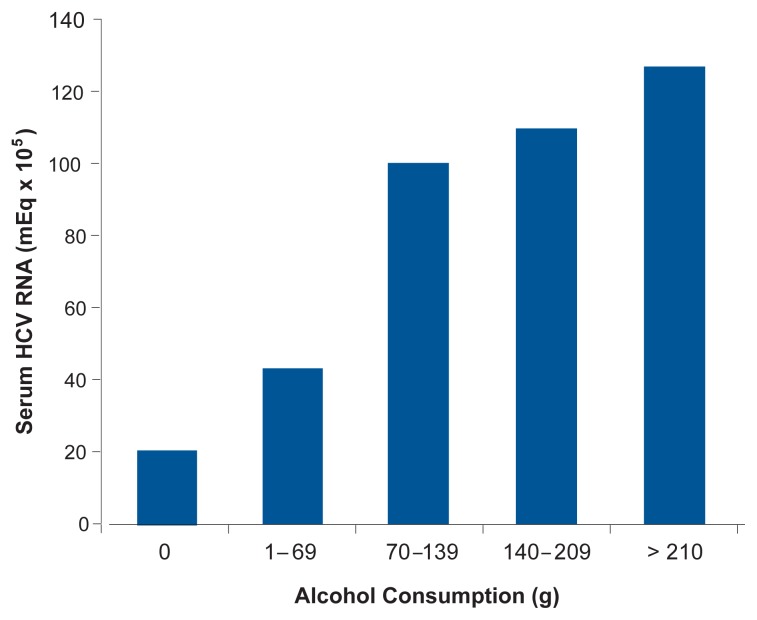
As (self-reported) alcohol consumption increases, mean blood HCV RNA levels also increased in patients with chronic HCV infection. SOURCE: Pessione et al. 1998.

**Figure 5:**
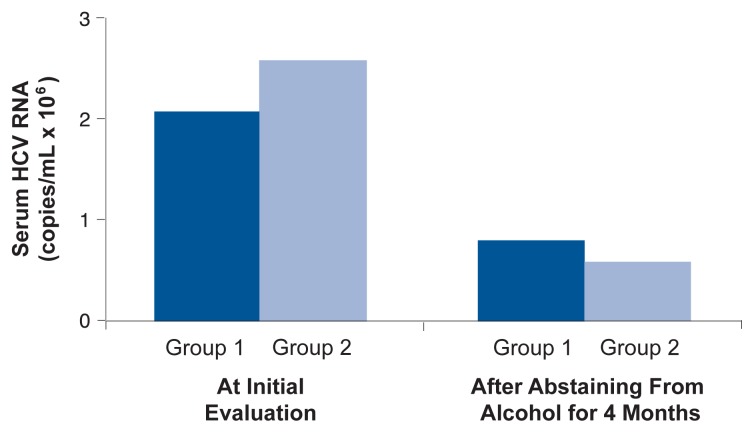
Reversibility of the increase in blood HCV RNA levels following a 4-month alcohol-free diet. Group 1 consisted of subjects who drank less than or equal to 10 grams (g) of alcohol per day when the study began; subjects in Group 2 consumed 10 g of alcohol or more per day at the outset of the study. SOURCE: Cromie et al. 1996.

Although researchers do not fully understand how alcohol consumption accelerates liver injury in patients with HCV infection, it is likely that several factors are involved. The following mechanisms have been proposed:

- Increased replication of HCV in the liver. As illustrated in figure 4, some research (although not all) has found that greater alcohol consumption is related to higher HCV RNA blood concentrations (Pessione et al. 1998). Moreover, as shown in figure 5, when people infected with HCV who drank more than 10 g of alcohol (about 5.8 drinks) per day abstained from alcohol or substantially reduced their consumption for 4 months before treatment, HCV RNA levels dropped; the decline in serum HCV RNA among subjects who drank less than or equal to 10 g of alcohol (less than one drink) per day before abstaining was not statistically significant (Cromie et al. 1996).
- Mutations of the HCV virus (forming what are known as quasi-species). Alcoholics infected with HCV show greater quasi-species complexity than do nonalcoholics with HCV infection. In alcoholic HCV patients, such increased viral complexity might make it difficult for the immune system to control the mutated viruses, leading to progressive injury (Takahashi et al. 2001).
- Increased programmed cell death (apoptosis) of liver cells. Apoptotic death of liver cells, which can ultimately lead to liver fibrosis, is increased by alcohol consumption in people with HCV infection (Szabo 2003).
- Higher levels of inflammation and immunoregulatory proteins (specifically, interleukin, tumor necrosis factor, and interferon). In research with mice, Geissler and colleagues (1997) noted that chronic alcohol feeding in mice inhibited immune responses (specifically, responses by T-helper cells and cytotoxic T-lymphocytes) that play a pivotal role in removal of HCV from the body (i.e., HCV clearance).
- Viral gene mutations.
- Fatty liver. Accumulation of fat in the liver is common in patients with HCV. Examining a large group of patients with HCV infection, Serfaty and colleagues (2002) found that fibrosis progressed about twice as quickly among drinkers with steatosis as among drinkers without steatosis or nondrinkers with or without steatosis.
- Accumulation of excess iron in body tissues (i.e., iron overload). Alcohol consumption increases iron stores in the liver, and iron overload seems to contribute to HCV disease progression by inducing fibrosis (Piperno et al. 1998).
- Oxidative stress. Alcohol stimulates the production of reactive oxygen-containing molecules (i.e., oxygen radicals). Heavy alcohol use also depletes the body’s supply of molecules that normally defend tissues against damage caused by oxygen radicals (i.e., antioxidants). This state, known as oxidative stress, may accelerate liver damage in patients with HCV (Rigamonti et al. 2003). (For more information about how alcohol use can lead to oxidative stress and subsequent liver injury, see the article by Nanji and French in the next issue of *Alcohol Research & Health,* [Vol. 27, No. 4].)
- Depression of the immune system by alcohol.

## Treatment Issues

Heavy alcohol use can be detrimental to HCV-infected patients’ long-term response to interferon therapy (see the sidebar for discussion of interferon treatment for HCV). It is likely that alcohol affects HCV treatment effectiveness both because drinking tends to interfere with patients’ adherence to therapy and because alcohol interferes with interferon therapy’s antiviral actions.

**Hepatitis C Infection: Disease Characteristics, Testing, and Treatment**

The symptoms and course of hepatitis C vary greatly. Many people who are infected with the virus show no symptoms (although they can still infect others), whereas some people may experience fatigue, weakness, fever, nausea, abdominal pain, poor appetite, muscle and joint pain, or yellowing of the skin and eyes (jaundice) (National Institutes of Health 2002). About 75 percent of patients who are infected with HCV develop chronic infection (Centers for Disease Control and Prevention 1998). Between 10 and 40 percent of HCV patients develop cirrhosis (a condition in which normal liver cells are replaced by scar tissue) within 20 to 40 years, and 1 to 3 percent develop liver cancer.

Many patients who develop cirrhosis show no symptoms of the disease and can expect long-term survival (Di Bisceglie 2000). Data from a cohort of European patients with cirrhosis followed for an average of 5 years showed that only 7 percent developed hepatocellular carcinoma, and 18 percent experienced symptomatic liver failure (Fattovich et al. 1997). There currently is no vaccine for hepatitis C.

***Testing and Treatment***

Blood tests can diagnose HCV infection, either by detecting antibodies to the virus or by detecting the presence and quantity of the virus’s genetic material (HCV RNA) itself (National Institutes of Health 2002). Liver biopsy is quite helpful for evaluating the disease’s severity prior to initiating treatment. Liver enzymes have little value in predicting fibrosis.

There are six known genetic variants (genotypes) of HCV, which vary geographically in their rate of occurrence. Genotypes 1 (which accounts for 70 to 75 percent of cases), 2, and 3 constitute the majority of genotypes in the United States.

The standard treatment for symptomatic hepatitis C infection is a combination of the drugs pegylated interferon (peginterferon) and ribavirin (National Institutes of Health 2002). Strict abstinence from alcohol is important during treatment.

Currently, the best indicator of effective treatment is a sustained virological response (SVR), defined by the absence of detectable virus 24 weeks after the end of treatment. Early viral response (a minimum 2 log decrease in viral load during the first 12 weeks of treatment) is predictive of SVR, and patients who fail to achieve an early viral response at week 12 should discontinue the treatment. Genotype 1 requires 1 year of treatment, whereas genotypes 2 and 3 require a 6-month course. In research with patients who did not consume alcohol during the therapy, SVR rates of 42 to 46 percent for genotype 1 and 76 to 82 percent for genotypes 2 and 3 have been obtained (Di Bisceglie and Hoofnagle 2002).

— Eugene R. Schiff and Nuri Ozden

ReferencesCenters for Disease Control and PreventionRecommendations for prevention and control of hepatitis C virus (HCV) infection and HCV-related chronic diseaseMorbidity and Mortality Weekly Reports4713919989790221Di BisceglieAMNatural history of hepatitis C: Its impact on clinical managementHepatology311014101820001073356010.1053/he.2000.5762Di BisceglieAMHoofnagleJHOptimal therapy of hepatitis CHepatology365 Suppl 1S12112720021240758510.1053/jhep.2002.36228FattovichGGiustinaGDegosFMorbidity and mortality in compensated cirrhosis type C: A retrospective follow-up study of 384 patientsGastroenterology1124634721997902430010.1053/gast.1997.v112.pm9024300National Institutes of HealthNational Institutes of Health Consensus Development Conference statement: Management of hepatitis C: 2002—June 10–12, 2002Hepatology365 Suppl 1S32020021240757210.1053/jhep.2002.37117

### Abstention Before Interferon Treatment

Some research has indicated that heavy drinkers who abstain from alcohol before interferon treatment respond better to treatment than do those who continue to drink. In one study, heavy drinkers who did not abstain from drinking before interferon treatment showed a total lack of HCV RNA clearance, whereas those who normally drank heavily but abstained from drinking before interferon treatment showed some improvement in HCV RNA clearance (and the virus completely disappeared in 15.8 percent of heavy drinkers who abstained before treatment) (Mochida et al. 1996).

**Figure 6:**
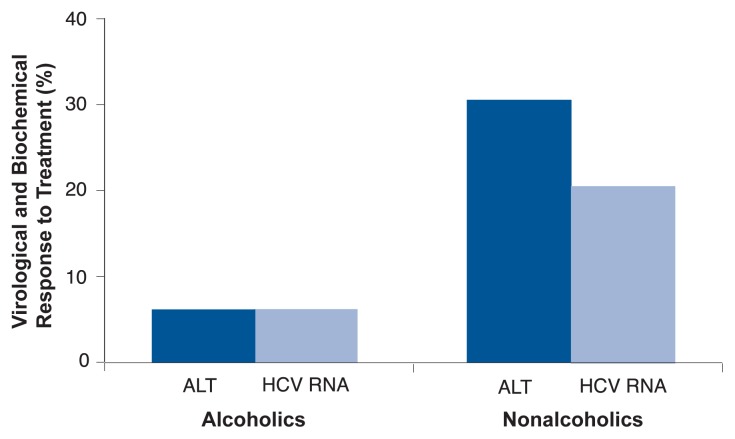
Six months after completion of a 24-week course of interferon treatment, HCV-infected patients who were not alcoholics showed significantly better virological and biochemical responses to therapy than did alcoholic patients. (Response to therapy was assessed by measuring blood levels of the virus itself [HCV RNA] and of the enzyme alanine aminotransferase [ALT], a marker for liver inflammation.) SOURCE: Mochida et al. 1996.

However, this study indicated that when HCV-infected alcoholic patients who normally drank heavily (70 or more grams of alcohol, or at least 5.8 drinks, per day) abstained from alcohol consumption during interferon therapy, their rate of HCV RNA clearance was markedly lower than was the case for HCV-infected nondrinkers (see figure 6). These results were confirmed by a recent Italian study (Tabone et al. 2002) in which 150 patients with chronic HCV underwent interferon treatment for 1 year after 6 months of abstinence from alcohol. (Abstinence was verified by analyzing blood levels of a biological marker of heavy alcohol consumption known as carbohydrate-deficient transferrin.) Although all patients had abstained from alcohol before beginning treatment, only 9 percent of (normally) heavy drinkers exhibited a sustained response to the interferon treatment, compared with 20 percent of light drinkers and 33 percent of nondrinkers. The factors that most strongly predicted a poor response to interferon treatment were the specific type of HCV infection (patients with HCV genotype 1b were least responsive), patients’ age, and their lifetime alcohol intake.

### Treatment Recommendations

The evidence is strong that continued heavy alcohol intake during interferon treatment adversely affects treatment effectiveness. Further, depression, irritability, and anxiety—side effects that occur in 20 to 30 percent of patients who receive interferon treatment— may be especially difficult to manage for patients with a history of alcoholism, predisposing them to begin drinking again (Zdilar et al. 2000). Despite this risk, however, the data do not support withholding interferon therapy for chronic HCV from patients with a history of alcoholism or heavy drinking if they remain abstinent and have adequate psychosocial support during treatment. Likewise, light-to-moderate drinkers should not be excluded from HCV treatment, nor should a period of abstinence before starting therapy be enforced in this patient population.

## Directions for Future Research

A recent National Institute on Alcohol Abuse and Alcoholism conference (Morgan et al. 2003) identified areas of research with the greatest potential for leading to more effective treatment options. Conference recommendations for research within these areas were as follows:

### Clinical Studies

- Determine how variations in the amount and pattern of drinking, combined drinking and smoking, and nutritional deficiencies affect HCV-infected patients’ risk of liver injury, disease progression, and death.
- Evaluate the effectiveness of alcohol cessation programs in patients with HCV.
- Specify how alcohol affects patients’ response to interferon treatment, including chemical interactions and day-to-day changes in virus activity during treatment.

### HCV Virology

- Determine how alcohol affects viral replication, clearance, and persistence and the evolution of new HCV quasi-species.
- Examine whether alcohol use leads to greater dominance of more harmful genetic variants of HCV.
- Determine whether alcohol interacts with the HCV viral proteins to alter the virus’s genetic activity.

### Immunology

- Identify the effects of alcohol on immune responses to HCV, including changes in quality, behavior, and survival of immune cell populations both within and outside the liver.

### Mechanisms of Liver Injury

- Determine what factors activate the liver cells that deposit fibrous material at the site of injury and cause scarring (i.e., hepatic stellate cells). These factors may include the patient’s immune response to HCV infection, the specific HCV genotype, and oxidative stress triggered by alcohol use.
- Identify factors (including complementary medicines) that may inhibit stellate cell activation and fibrosis in HCV-infected patients who use alcohol.
- Clarify the roles that alcohol and HCV infection play in oxidative stress, lipid peroxidation, and disruption of cell function.
- Determine the source of proteins, known as cytokines, that act as antibody-mediated (humoral) messengers between liver cells, and their role in liver cell injury and regeneration in HCV-infected patients who use alcohol.
- Evaluate the effect of alcohol and HCV infection on fatty liver and determine how steatosis contributes to liver disease.
- Determine how alcohol use and HCV infection influence various types of liver cells (i.e., hepatocytes, Kupffer cells, endothelial cells, and hepatic stellate cells).
- Characterize the effect of human liver cells (i.e., hepatocytes) on overall human pathophysiology in response to alcohol and HCV.
- Identify genetic factors that affect the severity of alcohol- and HCV-induced liver disease.

### Model Systems

- Develop animal models (using living animals and laboratory cell cultures) of HCV infection and of alcoholic liver disease that reproduce disease processes found in humans.

## Summary

Excessive alcohol consumption among patients infected with chronic hepatitis C is likely to result in more severe liver injury, promoting cirrhosis and increasing the risk for development of liver cancer (specifically, hepatocellular carcinoma). Although the mechanisms by which chronic hepatitis C progresses to more severe liver disease in alcoholic patients have not been clearly established, they may include an alcohol-induced increase in viral replication; rapid mutation of HCV, leading to greater viral complexity; increased liver-cell death and inflammatory response; suppression of immune responses; accumulation of fat in the liver; and accumulation of excess iron in body tissues.

In addition to greatly heightening HCV-infected patients’ risk of serious liver disease, heavy drinking during antiviral (interferon) treatment has been shown to impede patients’ responses to therapy. Abstaining from drinking before and during treatment improves patients’ response to antiviral therapy, although this improvement is not total. In light of these findings, alcoholic patients should be advised to abstain from further alcohol consumption and should be alcohol free for at least 6 months before beginning interferon therapy.

Future research should further examine the effects of light and moderate drinking on features of the hepatitis C virus; alcohol’s effect on virus activity and response to treatment; patients’ immune responses to the virus; mechanisms of fibrosis in response to HCV infection and alcohol use; and animal models of HCV infection that may shed greater light on disease processes in humans.

## Footnotes

1 The National Institute on Alcohol Abuse and Alcoholism (NIAAA) defines a standard drink as 11–14 g of alcohol. This corresponds to approximately one shot of 80-proof alcohol (about 14 g alcohol), one glass of wine (11 g), or one 12-oz. beer (12.8 g).

2 In these studies, definitions of moderate drinking differ from current Federal guidelines, which consider moderate drinking to be no more than one drink per day for women, and no more than two drinks per day for men (U.S. Department of Agriculture and the U.S. Department of Health and Human Services 1995).

## References

[b35-232-239] Centers for Disease Control and Prevention (1998). Recommendations for prevention and control of hepatitis C virus (HCV) infection and HCV-related chronic disease. Morbidity and Mortality Weekly Reports.

[b36-232-239] Di Bisceglie AM (2000). Natural history of hepatitis C: Its impact on clinical management. Hepatology.

[b37-232-239] Di Bisceglie AM, Hoofnagle JH (2002). Optimal therapy of hepatitis C. Hepatology.

[b38-232-239] Fattovich G, Giustina G, Degos F (1997). Morbidity and mortality in compensated cirrhosis type C: A retrospective follow-up study of 384 patients. Gastroenterology.

[b39-232-239] National Institutes of Health (2002). National Institutes of Health Consensus Development Conference statement: Management of hepatitis C: 2002—June 10–12, 2002. Hepatology.

